# Cavitary Pulmonary Disease Caused by *Mycobacterium kyorinense* : A Case Report on a 3‐Year Clinical Course

**DOI:** 10.1002/rcr2.70142

**Published:** 2025-03-05

**Authors:** Masahiro Yanagi

**Affiliations:** ^1^ Department of Respiratory Medicine Muroran City General Hospital Muroran Japan

**Keywords:** cavitation, *Mycobacterium kyorinense*, nontuberculous mycobacteria, sputum culture

## Abstract

An 80‐year‐old female presented with haemoptysis, and chest computed tomography (CT) revealed bronchiectasis in the right middle lobe accompanied by granular and nodular opacities in the right lower lobe. Although acid‐fast bacilli (AFB) smears were consistently positive, polymerase chain reaction (PCR) assays for *Mycobacterium tuberculosis* yielded negative results, and repeated sputum cultures on conventional solid‐phase media failed to identify the causative organism. Cavitary pulmonary lesions developed progressively over 3 years, accompanied by persistent AFB smear positivity. Ultimately, liquid culture using the Mycobacteria Growth Indicator Tube (MGIT) system isolated nontuberculous mycobacteria, subsequently identified as *Mycobacterium kyorinense* ( *M. kyorinense* ) via matrix‐assisted laser desorption/ionisation time‐of‐flight mass spectrometry (MALDI‐TOF MS). This case illustrates the pathogenic potential of *M. kyorinense* to induce cavitary pulmonary disease in otherwise healthy individuals, underscoring the necessity of early diagnostic strategies and prompt therapeutic intervention to prevent irreversible pulmonary damage.

## Graphical abstract

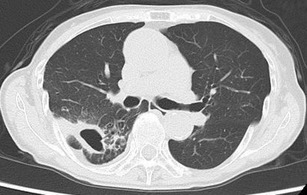

## 1 Introduction

Nontuberculous mycobacterial infections encompass diverse species, each demonstrating distinct clinical trajectories and necessitating tailored therapeutic approaches. Identifying infections caused by rare species presents a significant challenge, often requiring sophisticated molecular methodologies to achieve precise species differentiation.

*Mycobacterium kyorinense* ( *M. kyorinense* ) is a non‐pigmented, slow‐growing, nontuberculous mycobacterium first identified in Japan in 2009 [1]. Approximately 20 cases have been reported, primarily in Japan. However, additional cases identified in Brazil, Australia, Saudi Arabia, and India suggest a broader yet under‐recognised global distribution [2, 3]. Due to its rarity, comprehensive knowledge of its clinical manifestations and disease progression remains limited. In this report, we delineate the clinical trajectory of *M. kyorinense* infection, which led to cavitary lung disease over 3 years, and discuss the diagnostic challenges associated with its bacterial detection.

## 2 Case Report

An 80‐year‐old female presented to our hospital with a 5‐day history of haemoptysis. Her medical history was notable for gastro‐oesophageal reflux disease and dyslipidaemia, with no history of smoking or significant pulmonary disease. She had resided in the same region her entire life and had no history of international travel.

**FIGURE 1:**
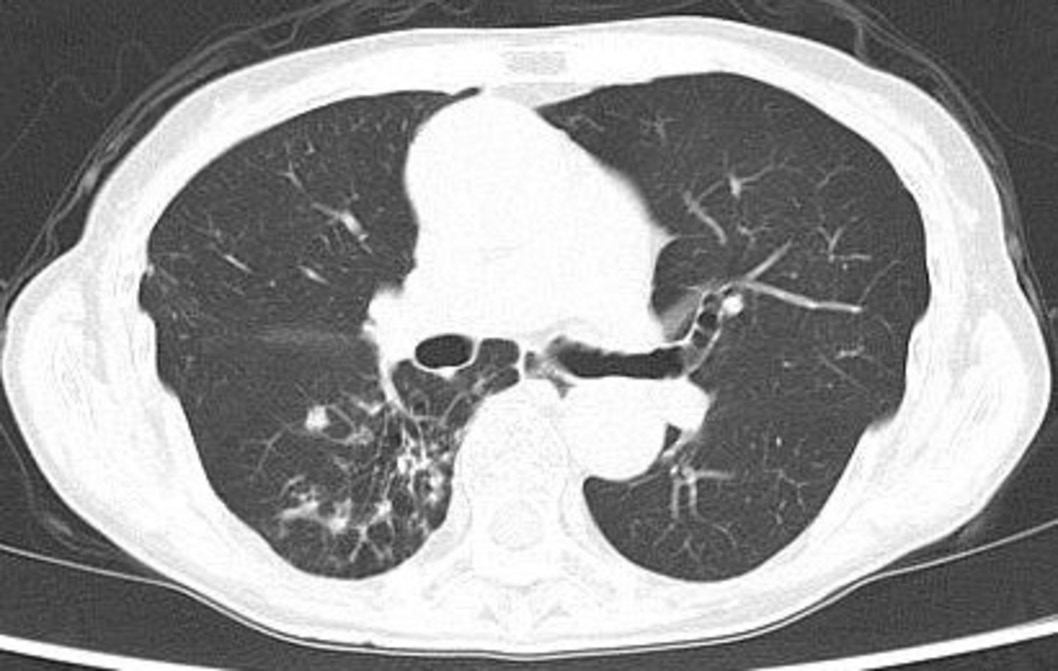
Chest computed tomography shows scattered granular and nodular opacities in the right lower lobe.

Chest computed tomography (CT) revealed bronchiectasis in the right middle lobe and scattered granular and nodular opacities in the right lower lobe (Figure 1). No lymphadenopathy, pleural effusion, or extrapulmonary lesions were noted. Laboratory investigations were unremarkable, with negative results for tuberculosis‐specific interferon‐gamma and anti‐*Mycobacterium avium complex* (MAC) antibodies and no evidence of immunodeficiency. Acid‐fast bacilli (AFB) smears of her sputum remained persistently positive (± or 1 +). However, polymerase chain reaction (PCR) assays for *Mycobacterium tuberculosis* ( *M. tuberculosis* ) and MAC were negative, and repeated sputum cultures on conventional solid media failed to identify the pathogen.

Although a rare nontuberculous mycobacterial infection was suspected, her haemoptysis resolved with haemostatic agents, and she declined further invasive diagnostic procedures, including bronchoscopy. Consequently, a follow‐up imaging strategy was adopted as the primary management approach.

**FIGURE 2:**
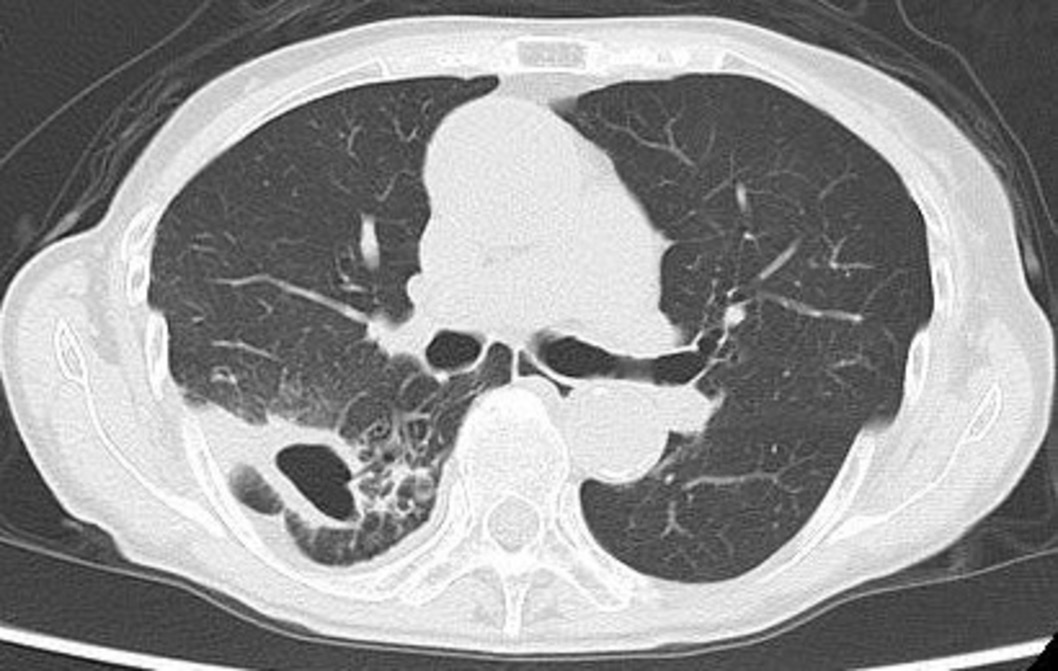
Chest computed tomography demonstrates a cavitary lesion formation in the right lower lobe.

Over 3 years, a cavitary lesion in the right lower lobe exhibited progressive enlargement (Figure 2). Repeat AFB smears remained persistently positive. However, solid media cultures failed to yield growth, preventing species identification. To overcome this diagnostic limitation, a liquid culture utilising the Mycobacteria Growth Indicator Tube (MGIT) method was performed, resulting in a positive detection of acid‐fast bacilli. Subsequent identification via matrix‐assisted laser desorption/ionisation time‐of‐flight mass spectrometry (MALDI‐TOF MS) confirmed the pathogen as *M. kyorinense* . The diagnosis was further substantiated by the isolation of *M. kyorinense* from an additional sputum specimen.

Despite the absence of symptoms such as cough, fever, or fatigue, the progressive enlargement of the cavitary lesion and the persistence of intermittent haemoptysis prompted consideration of pharmacological treatment guided by drug susceptibility testing (Table 1). However, the patient elected to decline treatment, and no further therapeutic interventions were pursued.

**TABLE 1:** Drug susceptibility testing results of *M. kyorinense* strains isolated from the patient.

Drug	MIC (μg/mL)
CAM	0.125
AZM	0.5
EB	4
KM	≤ 2
INH	4
RFP	> 4
RBT	0.5
MFLX	≤ 0.25
TH	1
MINO	2
DOXY	8
LZD	4
STFX	0.25
AMK	≤ 8

Abbreviations: AMK, amikacin; AZM, azithromycin; CAM, clarithromycin; DOXY, doxycycline; EB, ethambutol; INH, isoniazid; KM, kanamycin; LZD, linezolid; MFLX, moxifloxacin; MINO, minocycline; RBT, rifabutin; RFP, rifampicin; STFX, sitafloxacin; TH, ethionamide.

## 3 Discussion

This case underscores the pathogenic potential of *M. kyorinense* , a rare, slow‐growing, nontuberculous mycobacterium first identified in Japan in 2009 [1], to independently induce cavitary lung lesions, even without pre‐existing pulmonary abnormalities. Despite its limited characterisation, the phylogenetic similarity of *M. kyorinense* to *Mycobacterium celatum* and *Mycobacterium branderi* presents a diagnostic challenge due to its slow growth [1, 2]. This report highlights its capacity to compromise normal lung architecture over a prolonged disease course, emphasising the critical role of advanced diagnostic modalities such as the MGIT liquid culture system and MALDI‐TOF MS in facilitating timely and accurate identification [2, 3].

A prior study characterising *M. kyorinense* infections indicates that this pathogen can cause pulmonary disease even in immunocompetent individuals. However, cavitary lesions are typically associated with pre‐existing cysts or structural lung abnormalities [3]. Notably, this case demonstrated that over a three‐year period, *M. kyorinense* could independently disrupt normal lung architecture and induce cavity formation, highlighting its clinical significance.

At our institution, mycobacterial cultures are routinely performed using solid media, specifically Ogawa medium, due to its cost‐effectiveness and practicality. While sputum smears consistently showed (±) to (1 +), a level typically sufficient for detecting *M. tuberculosis* or MAC infections, repeated cultures on solid media failed to yield growth, delaying pathogen identification. In contrast, the MGIT liquid culture system offers superior sensitivity and faster detection than solid media. However, its implementation is associated with increased costs and necessitates stringent contamination control. In this case, early utilisation of the MGIT system might have enhanced diagnostic efficiency and facilitated the timely identification of the slow‐growing *M. kyorinense* .

Due to the limited number of reported cases, no standardised treatment protocol for *M. kyorinense* has been established. Previous studies consistently indicate that conventional anti‐tuberculosis medications, including isoniazid (INH), rifampicin (RFP), and ethambutol (EB), are ineffective [4]. Resistance to RFP is attributed to a mutation at Ser531 in the *rpoB* gene, a mechanism analogous to that observed in rifampicin‐resistant *M. tuberculosis* [4]. Favourable clinical outcomes have been documented with regimens incorporating macrolides (e.g., clarithromycin [CAM]), fluoroquinolones (e.g., moxifloxacin [MFLX]), and aminoglycosides (e.g., amikacin [AMK]), guided by MIC testing when employed as first‐line therapeutic agents [3, 4, 5]. In the present case, MIC testing revealed high resistance to INH and RFP, while low MIC values for CAM, MFLX, and AMK suggested their potential efficacy as first‐line agents.

In conclusion, this case illustrates the ability of *M. kyorinense* to cause cavitary pulmonary disease over a few years, even in immunocompetent individuals. Early implementation of liquid culture systems such as MGIT and prompt therapeutic interventions guided by susceptibility testing is critical for managing this rare infection.

## Author Contributions

The author contributed to the conceptualisation and design of the study, as well as to the acquisition, analysis, and interpretation of the data.

## Ethics Statement

The author confirms that written informed consent was appropriately obtained for the publication of this manuscript and its accompanying images.

## Conflicts of Interest

The author declares no conflicts of interest.

## Data Availability

The data that support the findings of this study are available on request from the corresponding author. The data are not publicly available due to privacy or ethical restrictions.
